# A Spirochaete is suggested as the causative agent of Akoya oyster disease by metagenomic analysis

**DOI:** 10.1371/journal.pone.0182280

**Published:** 2017-08-03

**Authors:** Tomomasa Matsuyama, Motoshige Yasuike, Atushi Fujiwara, Yoji Nakamura, Tomokazu Takano, Takeshi Takeuchi, Noriyuki Satoh, Yoshikazu Adachi, Yasushi Tsuchihashi, Hideo Aoki, Kazushi Odawara, Shunsuke Iwanaga, Jun Kurita, Takashi Kamaishi, Chihaya Nakayasu

**Affiliations:** 1 Research Center of Fish Diseases, National Research Institute of Aquaculture, Japan Fisheries Research and Education Agency, Minami-Ise, Mie, Japan; 2 Research Center for Bioinformatics and Biosciences, National Research Institute of Fisheries Science, Japan Fisheries Research and Education Agency, Yokohama, Kanagawa, Japan; 3 Marine Genomics Unit, Okinawa Institute of Science and Technology Graduate University, Onna, Okinawa, Japan; 4 Department of Biological production science, College of Agriculture, Ibaraki University, Ami, Ibaraki, Japan; 5 Mie Prefecture Fisheries Research Institute, Shima, Mie, Japan; 6 Ehime Prefecture Fisheries Research Center, Uwajima, Ehime, Japan; 7 Nagasaki Prefectural Institute of Fisheries, Nagasaki, Nagasaki, Japan; Bigelow Laboratory for Ocean Sciences, UNITED STATES

## Abstract

Mass mortality that is acompanied by reddish browning of the soft tissues has been occurring in cultured pearl oyster, *Pinctada fucata martensii*. The disease is called Akoya oyster disease (AOD). Although spreading pattern of the disease and transmission experiments suggest that the disease is infectious, the causative agent has not yet been identified. We used shotgun and 16S rRNA-based metagenomic analysis to identify genes that are present specifically in affected oysters. The genes found only in diseased oysters were mostly bacterial origin, suggesting that the causative agent was a bacterial pathogen. This hypothesis was supported by the inhibition of AOD development in naïve oysters injected with the hemolymph of diseased animals followed immediately with penicillin bath-administration. Further analyses of the hemolymph and mantle specifically and universally detected genes of bacteria that belong to phylum Spirochaetes in diseased pearl oysters but not in healthy oysters. By *in situ* hybridization or immunostaining, a *Brachyspira*-like bacterium was observed in the smears of hemolymph from affected oysters, but not from healthy oysters. Phylogenetic analysis using 16S rRNA sequences showed that the presumptive causative bacterium was outside of but most closely related to family Brachyspiraceae. We propose ‘Candidatus *Maribrachyspira akoyae*’ gen. nov, sp nov., for this bacterium.

## Introduction

Since the establishment of the culture pearl industry in the early nineteenth century, Japanese Akoya pearl had been considered as superior among cultured pearls [1]. However, mass mortality events of Akoya pearl oysters (*Pinctada fucata martensii*) marked the end of the golden age of Japanese pearl production. A mass mortality event in 1994 greatly reduced the quantity and quality of Akoya pearl [2], and in 1996 and 1997, the annual mortality of Akoya oysters was more than 50% of the oysters in production [3] with localized losses of about 80% [4]. In 1999, mother pearl oyster production in Japan was reduced to 15.6% of the 1989 levels [5].

In healthy pearl oysters, the adductor muscle and mantle is creamy white, but diseased oysters in mass mortality events exhibit reddish-brown coloration of the adductor muscle and mantle from summer to autumn [3, 6]. Histopathological changes in diseased oysters are characterized by marked infiltration of hemocytes and a collapse of the loose connective tissue [6]. Enlarged cells and the presence of granules and vacuoles in the cytoplasm of hemocytes were observed in diseased oysters [7].

The disease state observed in mass mortality events could be reproduced in healthy oysters following transplantation of pieces of the mantle from diseased pearl oysters into healthy oysters or through cohabitation with diseased oysters [6]. Injection of diseased oyster hemolymph also caused disease [8]. Thus, these mass mortality events were demonstrated to be due to infectious disease agent, and the disease was named Akoya oyster disease (AOD).

The causative agent is thought to be a virus or small bacterium, on the basis of demonstrated infectivity of 0.45 μm filtrated hemolymph (S1 Table). The causative virus was reportedly isolated from fish cells [3], but no other group successfully isolated the virus [9, 10]. Further, no presumptive causative virus had been detected by transmission electron microscopy (TEM) in diseased pearl oysters [11, 12]. Thus, the true nature of the causative agent for AOD has remained uncertain.

Metagenomic analyses using next-generation sequencing approaches have typically been used for pathogen identification in clinical [13–15]and environmental samples [16, 17]. Because various microorganisms are present in diseased oysters, the metagenomic approach is suitable for searches for the causative agent of AOD. First, we subjected the hemolymph of diseased pearl oysters to ultracentrifugation in order to make rough fractionations to identify the causative agent of AOD. Then, shotgun-metagenome analysis was performed against the ultracentrifuged fraction, and 16S rRNA-targeted metagenomic analysis was performed to compare the composition of bacterial communities in diseased and healthy oysters. On the basis of these findings, the causative agent of AOD was proposed to be a bacterium in phylum Spirochaetes. Observations of microbial cells by *in situ* hybridization and immunostaining, along with phylogenetic analysis using near full-length 16S rRNA sequences were used to propose that the pathogenic bacteria is in a new family.

## Materials and methods

### Ethics statement

No specific permits were required for the described field studies: a) no specific permissions were required for these locations/activities; b) location are not privately-owned or protected; c) the field studies did not involve endangered or protected species.

### Animals

**Fig 1:**
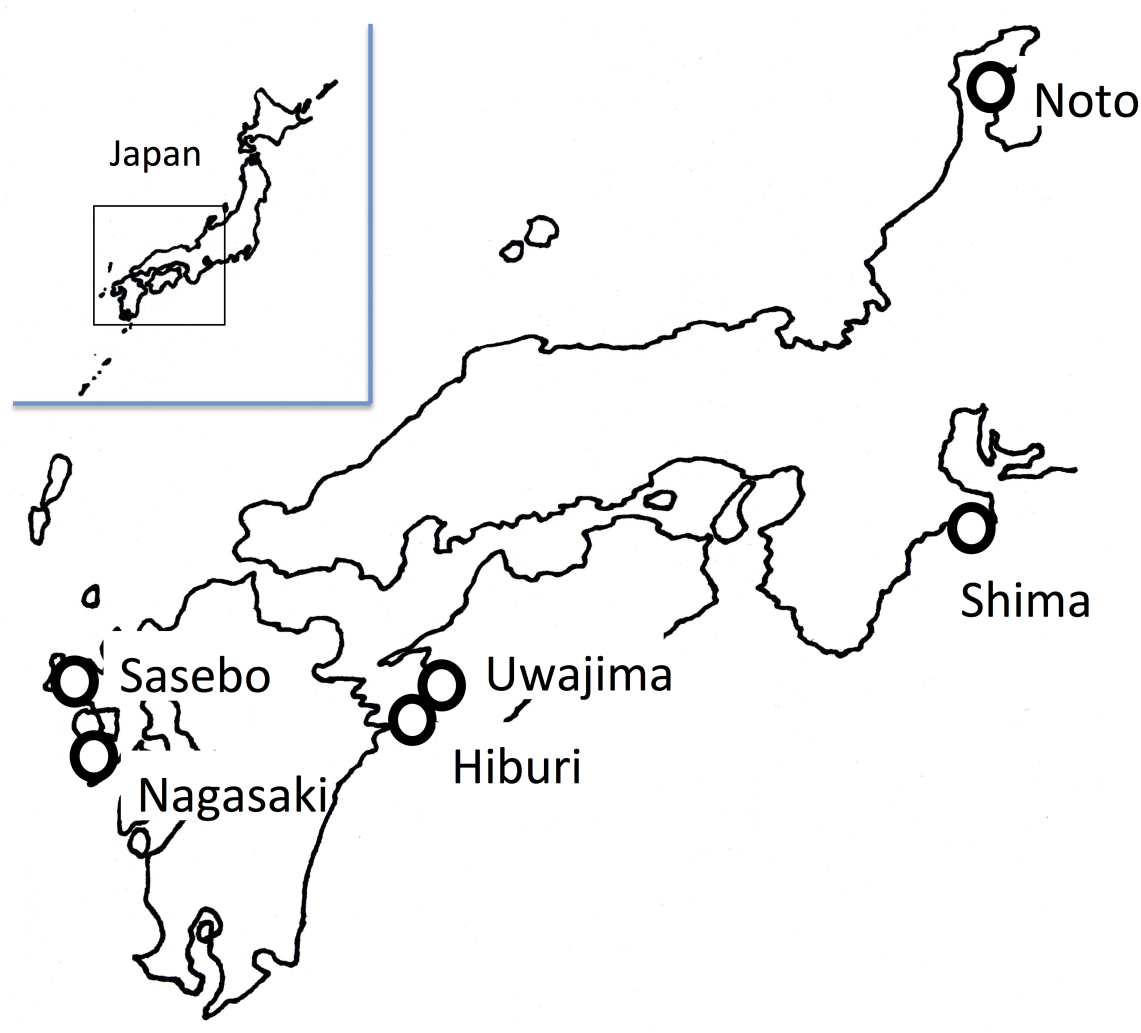
Sites of Akoya pearl oyster collection (open circles).

Diseased pearl oysters were collected from Uwajima (Ehime Prefecture), Shima (Mie Prefecture), and Sasebo (Nagasaki Prefecture) (Fig 1). These shells showed typical disease condition, such as change in color of the adductor muscle to reddish-brown and histopathological changes in the mantle and adductor muscle [6]. Healthy pearl oysters were collected from three areas free of AOD [18]: Noto (Ishikawa Prefecture), Hiburi (Ehime Prefecture), and Nagasaki (Nagasaki Prefecture). No signs of the disease were found on healthy pearl oysters. In the sampling for metagenomic analysis, pearl oysters were sacrificed upon arrival at the National Research Institute of Aquaculture. Healthy pearl oysters collected from Noto for infection in the laboratory were reared in 56 L tanks with running seawater controlled at 23–26°C and treated with UV light and filtration with a 1 μm pore size at the National Research Institute of Aquaculture. Pearl oysters were fed cultured diatom, *Chaetoceros calcitrans*, five times per week.

### Characterization of disease state

Adductor muscles were excised, and the degree of reddish-brown tincture was measured using a Chroma Meter CR-300 (Konica Minolta, Tokyo, Japan) and assigned an a-value on a Hunter color difference scale [19]. Abnormal reddish-brown tincture was clearly recognizable by eye when the a-value was over 3.0 [5], and an a-value above 4.0 was judged to be AOD [12].

### Determining the ultracentrifugation settings for fractionation of diseased oyster hemolymph

To determine the appropriate centrifugal force for fractionation, hemolymph was drawn from the adductor muscle of diseased pearl oysters obtained from Uwajima with 23-gauge needles. The average a-value of adductor muscle (number of oysters) was as follows for each experiment: experiment 1, 8.49±2.59 (n = 12); experiment 2, 5.00±2.91 (n = 12); and experiment 3, 5.03±2.44 (n = 9). The supernatant of the hemolymph collected by centrifugation at 1200 ×g for 5 min was pooled and ultracentrifuged for 60 min at 4°C using a SW40Ti rotor (Beckman-Coulter, Brea, CA) at the following settings: experiment 1, 50,000, 100,000, 190,000, and 290,000 ×g; experiment 2, 20,000, 30,000, 40,000, and 50,000 ×g; and experiment 3, 6,000, 10,000, 14,000, 17,000, and 20,000 ×g. After each centrifugation, the supernatant was collected, the sediment was resuspended in autoclaved seawater to the original volume, and the supernatant and resuspended sediment samples were injected into the adductor muscles of healthy pearl oysters obtained from Noto (100 μl/individual) with 23 × 1/4 gauge needles. In each experiment, the positive control group was injected with non-ultracentrifuged supernatant of the pooled hemolymph obtained from diseased oysters. The negative control group was injected with the non-ultracentrifuged supernatant of the pooled hemolymph obtained from healthy oysters. Mortality was monitored for 200 days in experiments 1 and 3 and for 180 days in experiment 2. The a-value was measured on the adductor muscle of surviving oysters.

### Sample preparation for shotgun metagenomic analysis

Sample groups were produced as follows. Hemolymph from seven diseased pearl oysters collected from Uwajima (a-value range 2.32–9.87; mean 5.66±2.32) was pooled and inoculated in twenty healthy Noto oysters. After 208 days, the hemolymph was collected from survived six oysters and assayed as the laboratory infection oyster group (LIOG) (a-value range 4.46–7.05, mean 5.52±1.1, at the time of sampling). Similarly, hemolymph of seven healthy pearl oysters collected from Noto (a-value range -1.53–1.02; mean 0.25±1.27) was pooled and inoculated in twelve healthy Noto oysters. After 208 days, the hemolymph was collected from six oysters that were randomly selected from seventeen survived oysters, and assayed as the healthy oyster group (HOG) (a-value range -0.76–2.16, mean 0.40±0.25, at the time of sampling).

Hemolymph was isolated from individuals in the LIOG and HOG as follows. The surfaces of the shells were scrubbed with autoclaved seawater, and oysters were carefully opened. Then, hemolymph was drawn from the adductor muscle, pooled for each group, and centrifuged at 1200 ×g for 5 min. An aliquot (12 mL) of each supernatant was ultracentrifuged at 20,000 ×g for 60 min. Sediments were re-suspended in 2.5 mL autoclaved seawater and divided into two portions. The first portion was placed in a 1.5 mL microcentrifuge tube and stored at -80°C until shotgun metagenomic analysis described below. The second portion was used for injections into healthy pearl oysters to confirm the presence of causative agent in the ultracentrifuged sediments as follows. Re-suspended sediments were injected into the adductor muscle of the healthy pearl oysters (100 μl/individual), and individuals of each group were placed in a tank (n = 20/tank) and incubated as described above. Mortality was monitored for 200 days.

### Sequencing for shotgun metagenomic analysis and data processing

To explore and identify the sequence(s) derived from the causative agent of AOD, two sequencing strategies were employed. Using longer sequencing reads increases resolution, making species identification possible. The 454 GS FLX+ platform (Roche, Basel, Switzerland) produces comparatively long reads and, therefore, we used this sequencer for the LIOG library. On the other hand, the HOG dataset was used to subtract the sequences of the resident (non-pathogenic) microorganisms from the LIOG dataset. For this purpose, having a larger number of sequencing reads from HOG is advantageous for effectively subtracting the sequences of resident microorganisms, and the HOG library was sequenced by the IonPGM™ platform (ThermoFisher Scientific, Waltham, MA), which gives much higher read numbers than the 454 GS FLX+ platform.

Metagenomic DNA was extracted from ultracentrifuged sediment by NucleoSpin Tissue XS (Takara Bio, Shiga, Japan) and amplified with a WGA2 kit (Sigma-Aldrich, St. Louis, MO), according to the manufacturer protocol. Amplified fragments were cleaned with Agencourt AMPure XP (Beckman-Coulter, Brea, CA) and sequenced at the National Research Institute of Fisheries Science.

**Fig 2:**
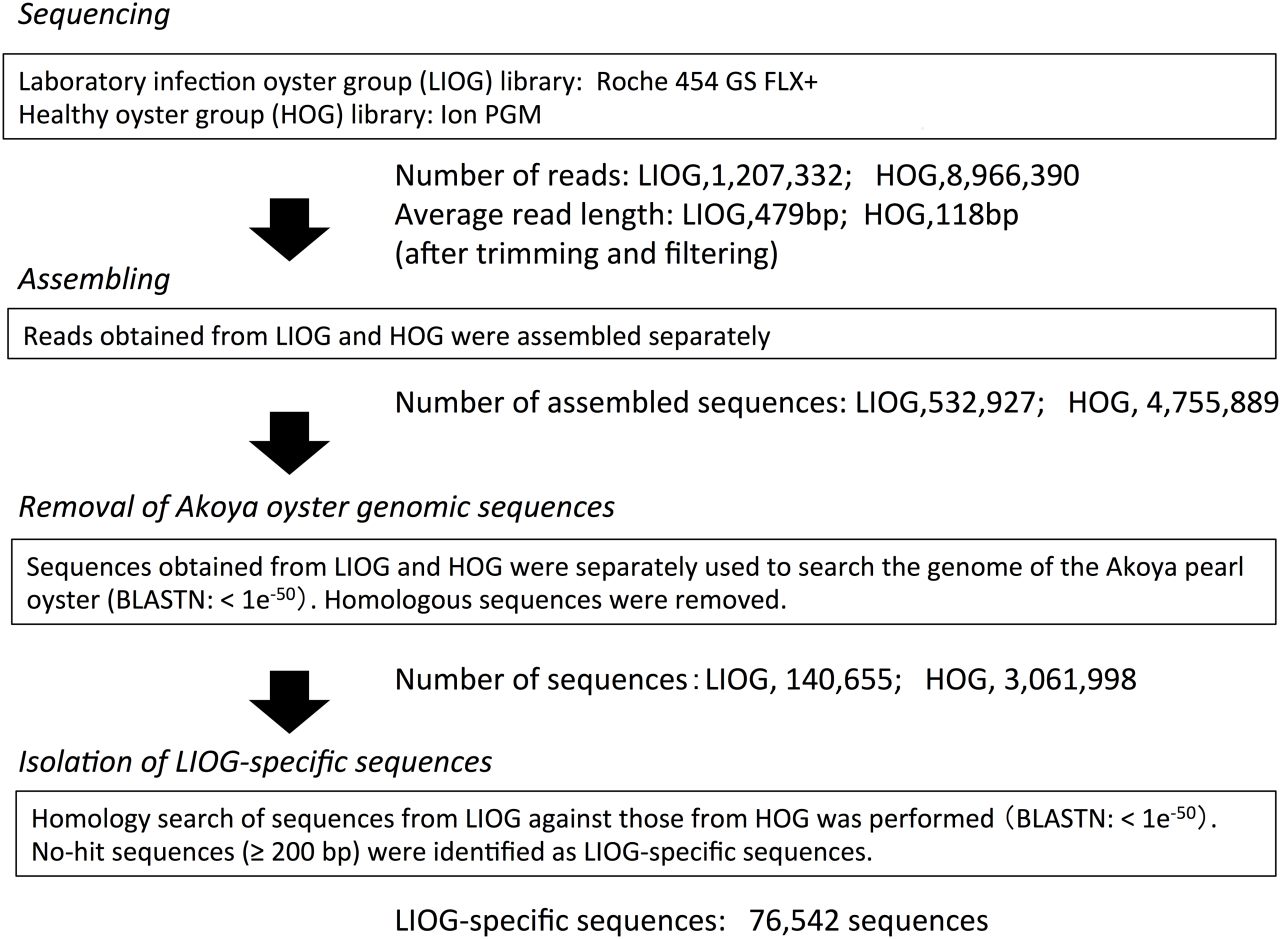
Strategy for analysis of shotgun metagenomic data.

Data processing was conducted as shown in Fig 2. Prior to *de novo* assembly, the sequence reads produced from both shotgun libraries (LIOG and HOG) were processed as follows. First, 30 bp was trimmed from either end of the raw sequence reads (30 bp + 30 bp = 60 bp), and the low quality sequences were filtered using CLC Genomics Workbench 5.5 (CLC Bio, Aarhus, Denmark) with default parameters (trimmed using quality scores = limit: 0.05, trim ambiguous nucleotides = maximum number of ambiguities: 2). *De novo* assembly for both LIOG and HOG datasets were conducted separately using CLC Genomics Workbench 5.5 with default parameters (de Bruijn graph (DBG) based assembly, word size = 20, bubble size = 50, minimum contig length = 200) (i.e., same assembly pipeline). Assembled sequences were homology searched using BLASTN (e-value < 1e^-50^) against the draft genome of Akoya pearl oyster [20] in order to remove host genomic DNA-derived sequences. The sequences obtained from LIOG were homology searched against the sequences obtained from HOG (BLASTN: <1e^-50^), and BLAST no-hit sequences were identified as LIOG-specific sequences. Prior to distinguishing the causative agent of AOD as a bacterium or virus, LIOG-specific sequences (≥200 bp) were subjected to BLASTN [21] searches against the NCBI nucleotide collection (nr/nt) database including both bacterial and viral genome sequences. This BLAST result was also utilized for detecting and eliminating contaminated eukaryotic genome sequences from the datasets. The best BLAST match (E value threshold of 1e^-4^) was used to identify closely related species. The abundances of each operational taxonomic units (OTU) were calculated by mapped read number against contigs, which were assigned to each OTU.

### Penicillin administration

Penicillin was administered as a bath to oysters injected with pooled hemolymph from diseased oysters. For use in inoculation, pooled hemolymph from diseased pearl oysters collected from Shima (n = 5; a-value range 0.81–8.80; mean 5.16±2.58) and pooled hemolymph from healthy pearl oysters collected from Noto (negative control; n = 5; a-value range -0.43–2.51; mean 1.32±1.03) were injected into 80 and 40 oysters collected from Noto, respectively. Oysters injected with hemolymph from diseased oysters were divided into four experimental tanks with static water. Two of the tanks were treated with penicillin G potassium (Wako, Osaka, Japan) at a final concentration of 50 U/mL. The positive control tanks were filled with clean, unmedicated water. For the negative control, the two experimental tanks were filled with clean, unmedicated water. Water and penicillin were changed every 24 h, and penicillin was bath-administered for 10 consecutive days. In all tanks, water was kept at 25°C using an electric heater during penicillin administration, and seawater controlled at 23–25°C was flowed through the tanks after penicillin administration. Mortality was monitored for 111 days, and five individuals were randomly selected from among the survivors at 111 days to measure the a-value in adductor muscles.

### Sampling for bacterial 16S rRNA gene-based metagenomic analysis

Hemolymph and mantle samples shown in Table 1 were characterized by 16S rRNA gene-based metagenomic analyses. The collected oysters were transported on ice to the laboratory, and upon arrival, the surfaces of the shells were scrubbed with autoclaved seawater to remove contamination. Oysters were carefully opened, and 200 μL of hemolymph was drawn from the adductor muscle. The mantle marginal zone was separated with sterile forceps, and the hemolymph and mantle were placed in 1.5 mL microcentrifuge tubes and stored at -80°C until further processing.

**Table 1:** Summary of sample characteristics for metagenomic analysis.

Tissue	Sample ID.	Pearl oyster disease state	Collection area	Collection date (YYYY.M.D)	Water temperature at collection (°C)	Number of pearl oysters	Shell length range (mm, mean±SD)	a-value (mean±SD)
*Hemolymph*								
	H1	healthy	Noto*	2012.6.12	25.0	6	63–82 (73.0±7.1)	0.02–1.91 (0.36±0.22)
	H2	healthy	Noto*	2013.10.28	24.5	1	73	-2.27
	H3	healthy	Noto*	2013.12.12	23.5	-	-	-
	H4	diseased	Uwajima	2011.11.16	17.2	7	65–80 (69±7.0)	4.5–12.32 (8.45±3.59)
	H5	diseased	Shima	2012.1.24	10.6	17	67–72 (69.4±2.25)	2.9–13.99 (6.02±2.76)
	H6#	diseased (AIOG)§	Artificial infection	2012.6.12	25.0	6	60–75 (67.0±4.7)	4.46–7.05 (5.52±1.1)
	H7	diseased	Shima	2012.9. 7	26.2	8	62–76 (72±5.2)	1.95–8.74 (4.07±2.32)
	H8	diseased	Uwajima	2012.11.6	20.1	1	80	4.02
	H9	diseased	Uwajima	2012.11.6	20.1	1	76	8.3
	H10	diseased	Uwajima	2013.12.3	15.0	1	65	6.9
	H11	diseased	Uwajima	2013.12.3	15.0	1	72	6.63
	H12	diseased	Uwajima	2013.12.3	15.0	1	68	5.81
*Mantle marginal zone*								
	M1	healthy	Noto*	2014.11.28	24.0	1	75	1.22
	M2	healthy	Noto*	2014.12.1	24.0	1	68	-0.11
	M3	healthy	Noto*	2014.12.10	24.0	1	67	0.21
	M4	healthy	Hiburi	2014.12.10	18.6	1	45	-1.78
	M5	healthy	Hiburi	2014.12.10	18.6	1	48	-0.41
	M6	healthy	Hiburi	2014.12.10	18.6	1	44	-1.16
	M7	healthy	Nagasaki	2014.11.11	20.4	1	73	1.04
	M8	healthy	Nagasaki	2014.11.11	20.4	1	65	2.68
	M9	healthy	Nagasaki	2014.11.11	20.4	1	64	1.01
	M10	diseased	Uwajima	2014.11.27	20.6	1	68	5.27
	M11	diseased	Uwajima	2014.11.27	20.6	1	72	4.91
	M12	diseased	Uwajima	2014.11.27	20.6	1	68	5.6
	M13	diseased	Uwajima	2014.11.27	20.6	1	77	2.24
	M14	diseased	Uwajima	2014.11.27	20.6	1	68	-0.18
	M15	diseased	Uwajima	2014.11.27	20.6	1	70	4.72
	M16	diseased	Shima	2014.11.27	16.1	1	72	0.12
	M17	diseased	Shima	2014.11.27	16.1	1	68	-0.13
	M18	diseased	Shima	2014.11.27	16.1	1	74	3.85
	M19	diseased	Sasebo	2014.11.11	20.2	1	60	2.4
	M20	diseased	Sasebo	2014.11.11	20.2	1	62	4.15
	M21	diseased	Sasebo	2014.11.11	20.2	1	62	4.22

*Healthy oysters collected at Noto were reared at the National Research Institute of Aquaculture for more than 6 months before use in experiments.

^#^Used for both shotgun metagenomic analysis and 16S rRNA gene cloning. Hemolymph from diseased oyster collected at Uwajima was injected into healthy oysters collected at Noto.

^§^ Laboratory infected oyster group

AOD had been eradicated from Hiburi and Nagasaki prior to sample collection [18].

AOD was never observed at Noto.

### DNA extraction, 16S rRNA gene library construction, pyrosequencing, and data processing

DNA was extracted with QIAamp DNA mini kit (Qiagen, Hilden, Germany) according to manufacturer protocol. Regions V1-V3 of 16S rDNA were amplified with the barcoded universal bacterial 27F (GAGTTTGATCMTGGCTCAG) and 518R (WTTACCGCGGCTGCTGG) primer pair. PCR reactions were performed by Macrogen Japan (Kyoto, Japan) based on the original protocol described before [22].

The quality of PCR products was checked on the Nanodrop (ThermoFisher Scientific). Briefly, equal amounts of each product obtained from 12 hemolymph samples and from 21 mantle samples were respectively pooled and cleaned using Agencourt AMPure XP. Pyrosequencing of each of the pooled hemolymph and mantle samples was performed by Macrogen Japan using the Roche GS-FLX454 platform. Data processing was performed using Roche GS FLX software (v2.9, Roche, Basel, Switzerland). The software uses tag (barcode) sequences to segregate reads in each sample by matching initial and final bases of reads to known tag sequences used in the preparation of libraries. Finally, sequences containing more than one ambiguous base (‘N’) were removed.

Sequencing data were analyzed using the pyrosequencing pipeline in the Silva rRNA database (http://www.arb-silva.de/). BLAST software was used with e = 0.01 while searching for the top five E-values (candidate hits). Then, global alignments of selected candidate hits were performed using the NEEDLE global alignment program [23] to determine the OTU. The taxonomic classification assigned to query was determined through the following steps using taxonomic information about the best hit. The relative abundance of members of a given phylum was defined as the total number of reads in the phylum divided by those in the entire bacteria community. Alpha and beta diversity indices were calculated using the BPMSG diversity calculator (http://bpmsg.com) with default parameters at the phylum level.

To analyze the variation among 16S rRNA sequences assigned to phylum Spirochaetes, the CAP3 program [24]was used to assemble 454 reads with custom set parameters (overlap percentage identity, 90%; overlap length, 450 bp). A total of 73,336 reads were classified as Spirochaetes and used for this analysis.

### 16S rRNA gene cloning, sequencing, and phylogenetic analysis

16S rRNA gene was amplified using universal bacterial 16S rRNA gene primers 20F (5′-AGAGTTTGATCMTGGCTCAG-3′, *Escherichia coli* positions 8–27) and 1500R (5′-GGTTACCTTGTTACGACTT-3′, *E*. *coli* positions 1509 to 1491) [25] with Ex *taq* (Takara Bio), according to manufacture protocol. The thermal profile consisted of an initial denaturation step of 95°C for 5 min followed by 30 cycles of 95°C for 30 s, 55°C for 30 s, and 72°C for 90 s, and then a cycle of 72°C for 7 min. A DNA sample for shotgun-metagenomic analysis was used for the PCR template (Table 1). The amplified fragment was ligated with the plasmid vector pCR4 TOPO TA vector (ThermoFisher Scientific), and the ligated DNA was transformed into *Escherichia coli* JM109. Plasmids were extracted from 48 randomly chosen colonies and sequenced using an ABI PRISM BigDye Terminator v3.1 Cycle Sequencing Kit (Applied Biosystems, Foster, CA) with M13 forward and reverse primers. Plasmids having insert sequences matched to the C10 sequence were selected, and the total length of the insert was analyzed using primers listed in S2 Table. A maximum-likelihood phylogenetic tree made using 17 sequences of the 16S rRNA gene, including type species for 10 Spirochaetes genera, type strains for 7 *Brachyspira* genera, and a sequence obtained in this study, was created using 1000 bootstrap replicates of the 16S rRNA sequence alignments in MEGA 7 [26]. Non-type strain *Spirochaeta stenostrepta* was included in the phylogenetic analysis because the 16S rRNA gene sequence of type species *S*. *plicatilis* [27] was not available.

### Fluorescent in situ hybridization (FISH)

To selectively identify causative bacterium, specific 16S rRNA directed FISH probe named AOD-Cy3 (5′-GTATTAATCCAATTTTCACT-3′, *E*. *coli* positions 151 to 170) was designed by modification with *Brachyspira* genus-specific probe [28]. A sense probe named non-AOD-Cy3 (AGTGAAAATTGGATTAATAC) was used as a negative control. The probe sequence was searched against 16S rRNA metagenomic sequences, and it was not identified from healthy pearl oysters. Probes were synthesized commercially and 5′ end labeled with a Cy3 (Fasmac, Kanagawa, Japan). FISH was performed using cytocentrifuge slides. Hemolymph was drawn from the adductor muscle of two diseased pearl oysters collected at Shima (a-value 6.56 and 7.20) and two healthy pearl oysters collected at Noto (a-value -0.45 and 1.37). Hemolymph was smeared onto a glass slides for 1 min at 1,000 rpm using a cytocentrifuge (Shandon Scientific Limited, Cheshire, UK) and then fixed in 4% paraformaldehyde at 4°C for 12 h. Fixed smears were dehydrated in an increasing series of ethanol and air-dried, and then incubated with 10 μg/mL concentration of lysozyme from egg white (Wako) in 100 mM Tris-HCl (pH7.6), 50 mM EDTA for 10 min at 37°C. After washing twice in distilled water, hybridization was performed as previous report [28]. Slides were mounted with Fluoro-Keeper mounting medium (Nacalai Tesque, Kyoto, Japan), and observed with a BX51 microscope (Olympus, Tokyo, Japan). Images were captured with an image processing system (VB-7000, Keyence, Osaka, Japan).

### Double-fluorescent immunostaining

Rabbit polyclonal antibodies against *Brachyspira aalborgi* NCTC11492, *B*. *pilosicoli* ATCC51139 [29], and *Treponema pallidum* (Abcam, Cambridge, UK) were labeled with Alexa555, and naïve rabbit antibody was labeled with Alexa488 by Zenon labeling kit (ThermoFisher). Hemolymph was drawn from three diseased pearl oysters collected at Shima (a-value 3.03, 6.21, 6.50) and three healthy pearl oysters collected at Noto (a-value 0.21, 1.22, 1.04), and smears were prepared as described above. After fixing with acetone for 1 min, slides were incubated with blocking buffer containing 5% naïve rabbit serum and 2% skim milk (Difco, Detroit, MI, USA) for 1 h at room temperature. Combinations of Alexa488- and Alexa555-labeled antibody (1:400 dilutions respectively) were applied to the smears. After incubation in a dark humid chamber at room temperature for 1 h, slides were rinsed with TBS. Images were captured as describe above. Size of the stained bacteria was measured using the FISH or immunostained smears with imageJ software (https://imagej.nih.gov/ij/).

### Statistical analysis

Data are presented as mean±standard deviation (SD). Cumulative mortality was statistically analyzed with Fisher’s exact tests. Mean a-value and the relative abundance of each OTUs were statistically analyzed by Wilcoxon signed-rank test. A significance level of *p*<0.05 was used. Alpha and beta diversity was statically analyzed by Student’s t test.

### Nucleotide sequence accession numbers

All sequences were deposited in the DDBJ with Accession Numbers DRA005313 and DRA005321 (shotgun metagenomic analysis), SAMD00070095-70127 (16S rRNA gene-based metagenomic analysis), and LC212988 (*16S rRNA gene*).

## Results

### Determining the ultracentrifugation settings for fractionation of diseased oyster hemolymph

**Fig 3:**
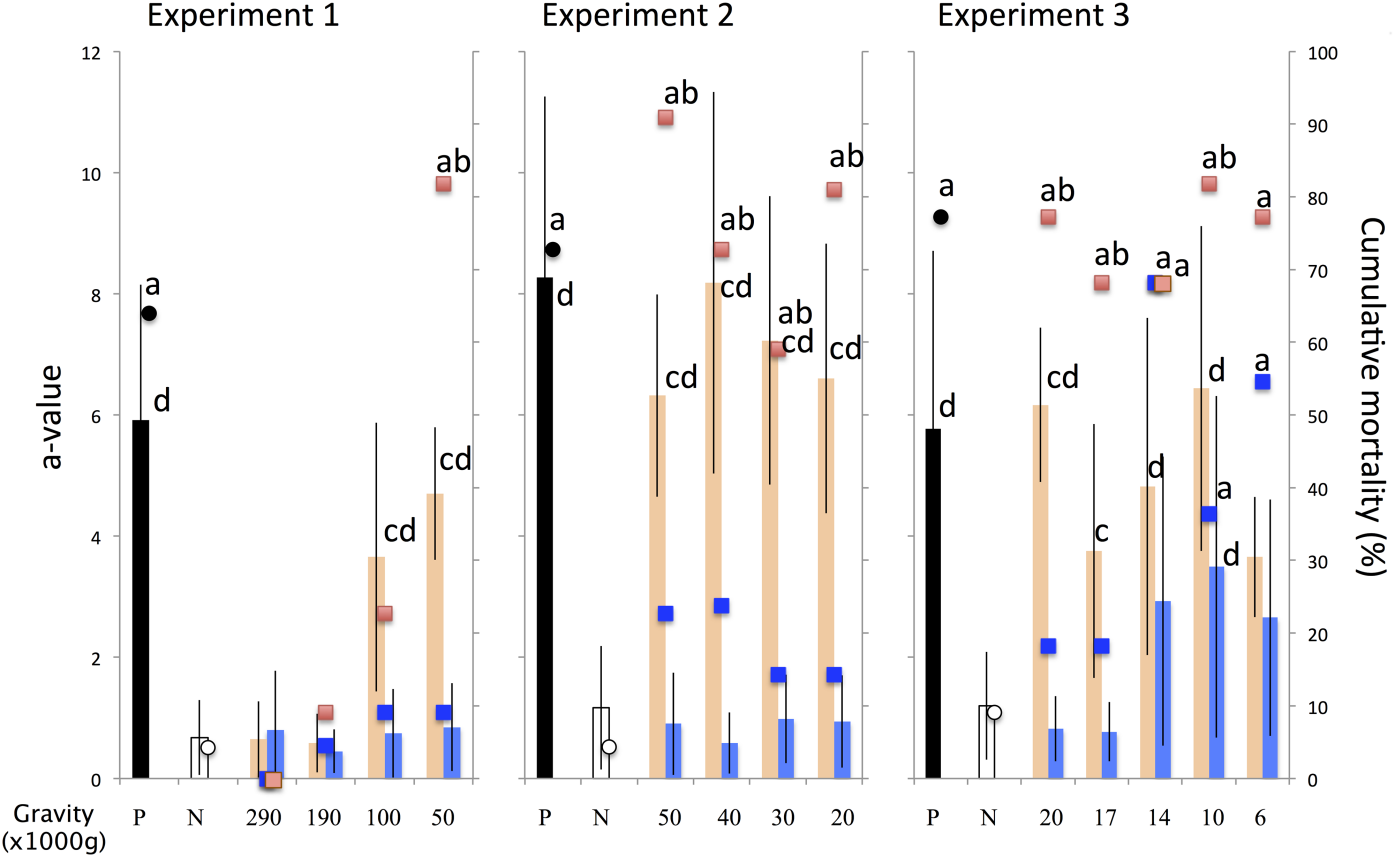
Survivor adductor muscle a-value (bars) and cumulative mortality (plotted points) are shown for each group. The groups are color coded as follows: positive control, black; negative control, white; sediment-inoculated groups, orange; and supernatant-inoculated groups, blue. Letters indicate statistically significant differences: a, cumulative mortality significantly different from the negative control (Fisher's exact test, p<0.05); b, cumulative mortality significantly different from the supernatant-injected group (Fisher's exact test, p<0.05); c, mean a-value significantly different from the supernatant control (Wilcoxon signed-rank test, p<0.05); and d, mean a-value significantly different from negative control (Wilcoxon signed-rank test, p<0.05).

For groups injected with inocula prepared by ultracentrifugation from 17,000 to 50,000 ×g, the cumulative mortality and a-value in survivors were significantly higher than those in supernatant-injected groups (Fig 3). These results indicate that the causative agent is contained in the fraction sedimented by ultracentrifugation from 17,000 to 50,000 ×g. Cumulative mortality and a-value in survivors were not significantly different in groups injected with inocula prepared by ultracentrifugation from 6,000 and 14,000 ×g, indicating that the causative agent was not separated in this fraction. Sediments obtained by ultracentrifugation from 190,000 and 290,000 ×g produced much lower mortality and survivors had a much lower a-value than for other test groups, and there were no differences between test and control (supernatant-injected) groups. The causative agent may have been inactivated by centrifugal force greater than 190,000 ×g.

### Shotgun metagenomic analysis

The presence of causative agent in samples used for shotgun metagenomic analysis was confirmed with an laboratory-infectied group. Briefly, cumulative mortality in groups injected with ultracentrifuged sediment from the hemolymph of LIOG and HOG was 70% and 10%, respectively (Fisher's exact test, *p*<0.05). The a-value of survivors, which were injected with ultracentrifuged sediment from the hemolymph of LIOG (n = 6; range, 7.00–7.65; mean±SD, 6.17±1.65) was significantly higher than that of survivors injected with ultracentrifuged sediment from the hemolymph of HOG (n = 5; range, -1.15–1.77; mean±SD, 0.60±0.79) (Wilcoxon signed-rank test, *p*<0.05). These results indicate the presence of a causative agent in the LIOG sample that is absent in the HOG sample.

**Fig 4:**
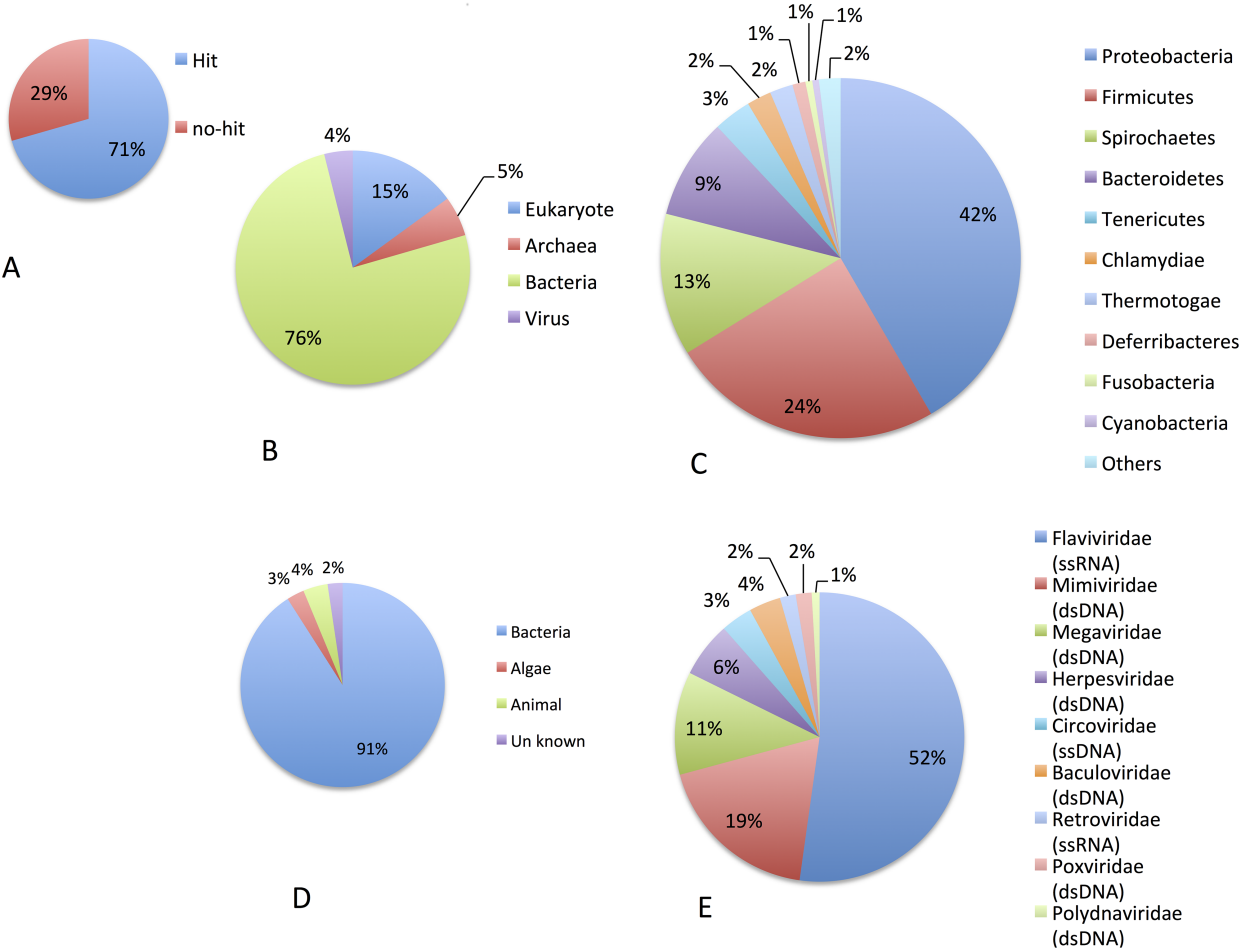
Relative abundance of identified reads in diseased oyster-specific sequences. (A) Reads with significant similarity to sequences in the GenBank nr database. (B) Distribution of reads with similarities among major biological groups. (C) Distribution of reads among 10 major Bacteria phyla. (D) Distribution of reads mapping to viruses by host taxon. (E) Distribution of reads mapping to animal viruses by virus type. Parentheses indicate the type of virus genome.

Sequencing of shotgun metagenomes obtained from LIOG and HOG hemolymph DNA samples and the subsequent data mining process (shown in Fig 3) produced a total of 76,542 sequences (≥200 bp) specific to LIOG. Summaries of BLAST homology searches are shown in Fig 4. Among the 76,542 LIOG-specific sequences, 34,098 sequences, consisting of 182,2991 reads (71% of the total LIOG-specific reads), were taxonomically classified by an E-value<10^−4^ (Fig 4A). The relative abundance of sequences found among these sequences showed that most sequences were classified as bacteria (76%, Fig 4B). Within kingdom Bacteria, phyla Proteobacteria, Firmicutes, Spirochaetes, Bacteroidetes, and Tenericutes account for 91% of the total reads mapped to this kingdom (Fig 4C). Within the domain Archaea, 93.7% of the reads are assigned to *Thaumarchaeota* (not shown). Classification of virus sequences by predicted host taxon matched over 97% of reads to bacteriophages. The number of reads identified as being animal virus was 113, which is 0.06% of the total BLAST reads with a hit in the GenBank nr database (Fig 4D). Reads mapping to animal viruses were categorized to the following families in order of *Flaviviridae*>*Mimiviridae*>*Megaviridae*>*Herpesviridae* (Fig 4E).

### Penicillin bath administration

**Fig 5:**
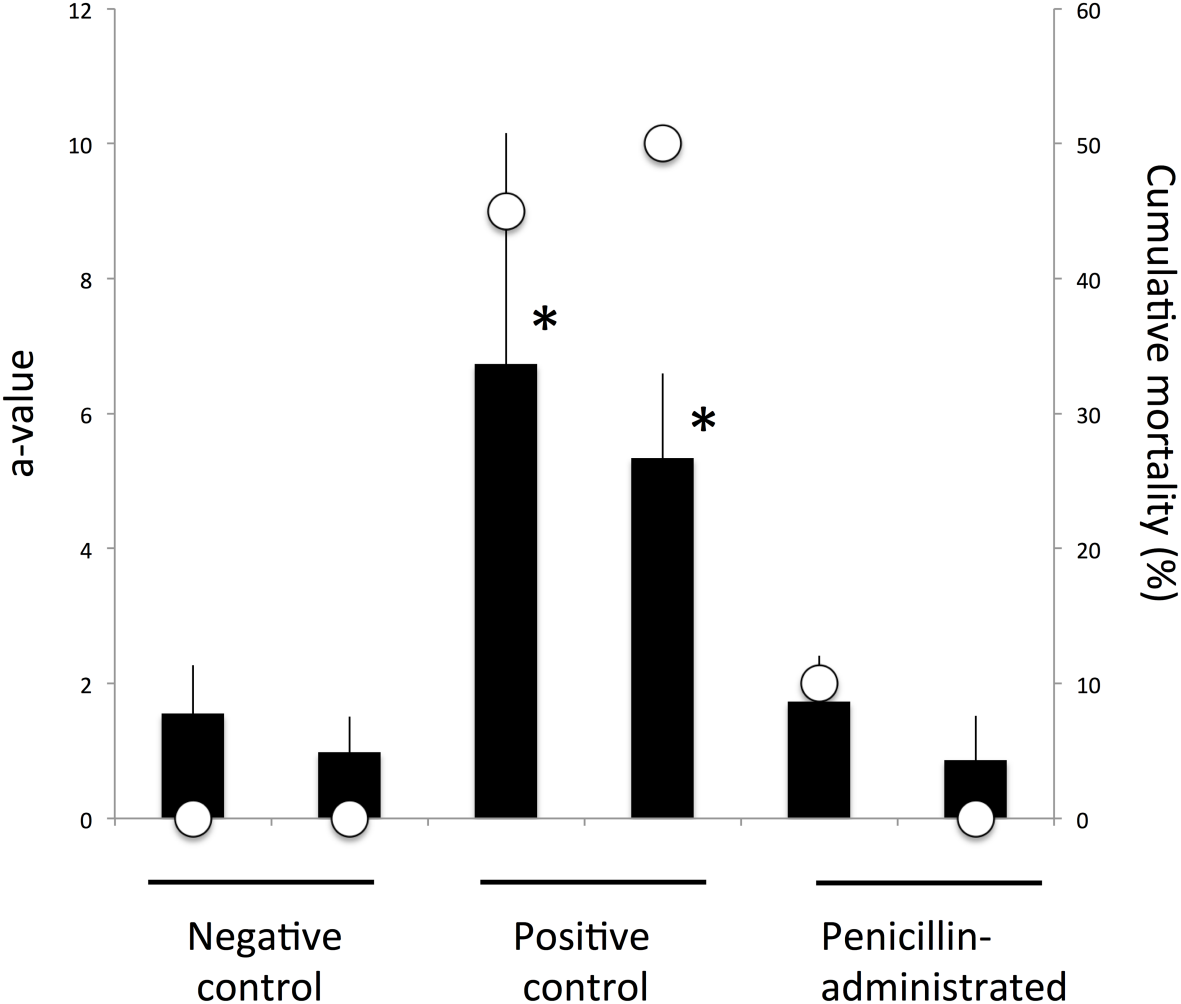
Effect of penicillin administration on cumulative mortality and a-value of the survivors. The a-value of the survivors is represented with bars and cumulative mortality is plotted with open circles. Asterisks indicate significant differences between negative control groups (Wilcoxon signed-rank test, *p*-value<0.05).

The cumulative mortality was 0% for both negative control groups, 45% and 50% for the positive control groups, and 10% and 0% for the penicillin-administered groups (Fig 5). The cumulative mortality of positive control groups was significantly higher than that of negative control groups and penicillin-administered groups (Fisher's exact test, *p*<0.05). The a-values of the positive control groups were significantly higher than those of the negative control groups and penicillin-administrated groups (Wilcoxon signed-rank test, *p*<0.05).

### 16S rRNA gene-based metagenomic analysis

**Fig 6:**
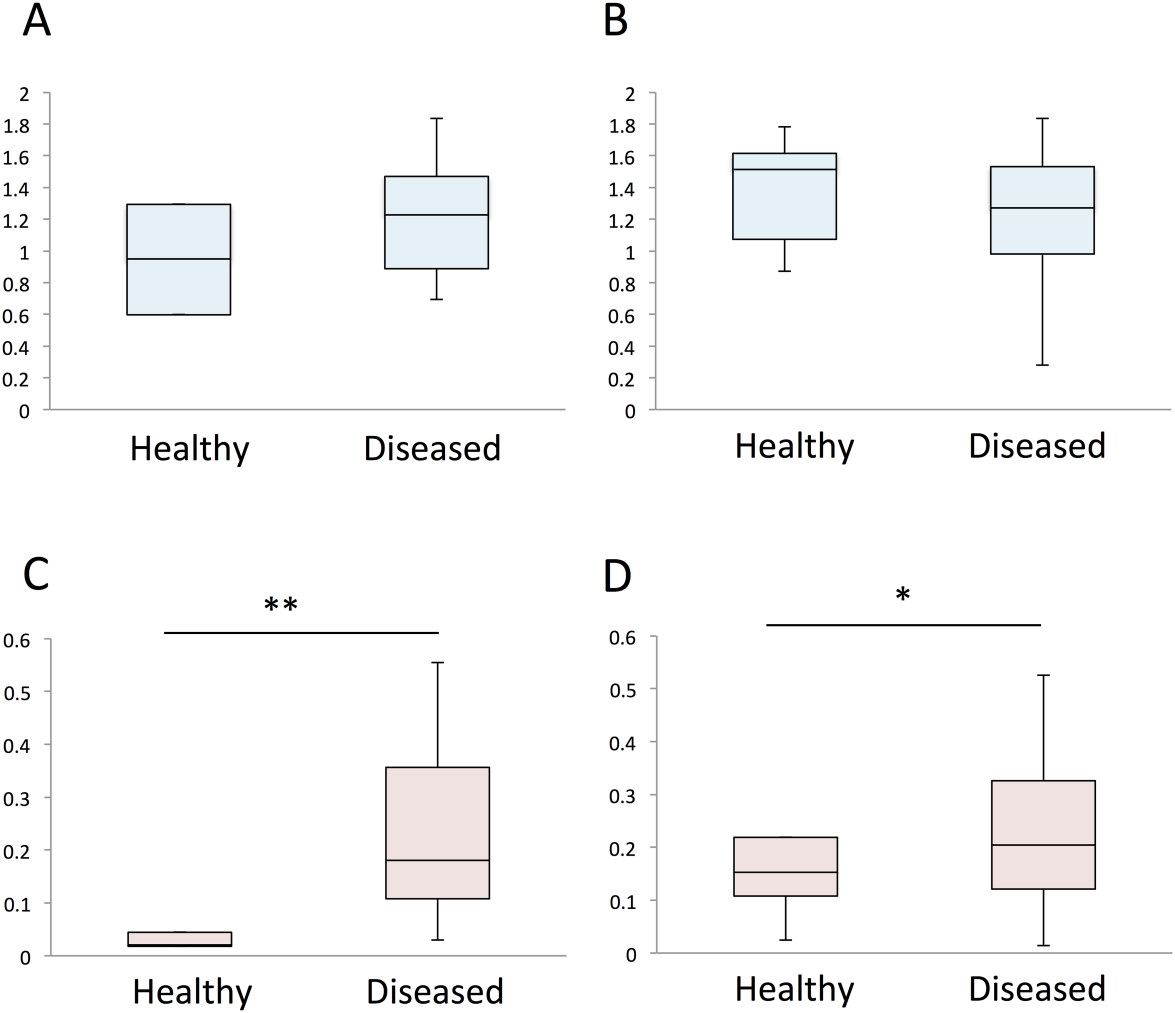
Bacterial diversity of the healthy and diseased pearl oysters. Shannon alpha (A, B) and Bray-Curtis beta (C, D) diversity of the hemolymph (A, C) and mantle (B, D) microbes were evaluated using OTUs at the phylum level. Box plots show median, 25th, and 75th percentiles, and whiskers represent minimum and maximum values. Student’s t test; *p<0.05; **P<0.01.

Comparative analysis of the diseased and healthy pearl oyster microbial communities revealed difference between the two groups. No difference in alpha diversity was observed between samples of healthy and diseased pearl oysters (Fig 6A and 6B). In terms of beta diversity, hemolymph and mantle bacterial communities in healthy oysters were more homogenous than those of diseased oysters (Fig 6C and 6D).

**Fig 7:**
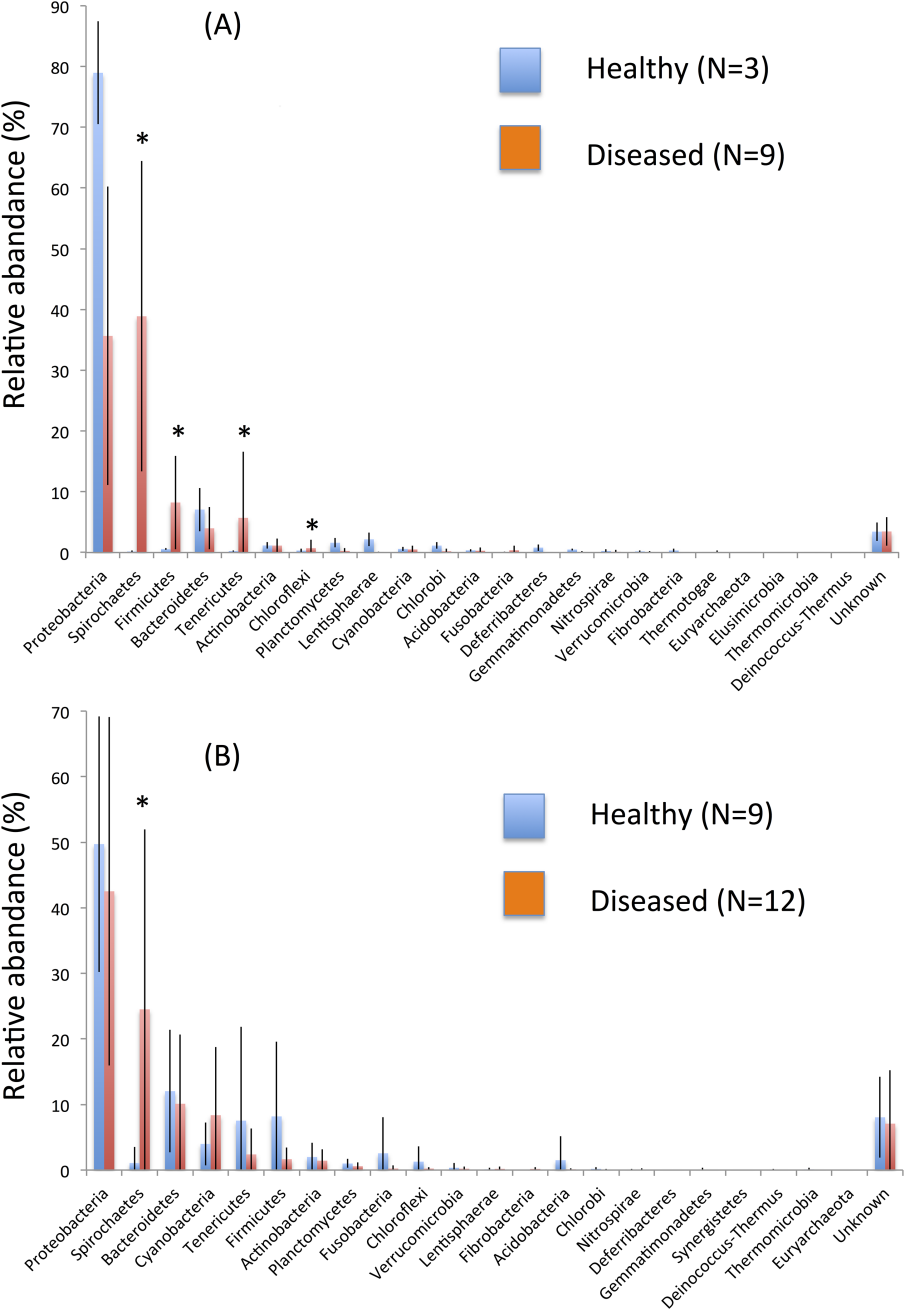
Normalized abundance distribution of each taxon at the phylum level from healthy and diseased pearl oyster in (A) hemolymph and (B) mantle. Percentage of total sequence reads in samples from healthy and diseased pearl oyster of hemolymph and mantle is presented. The error bars show calculated standard deviation of the mean. Asterisks indicate significant difference between relative abundance for healthy and diseased oysters (Wilcoxon signed-rank test, *P*-value<0.05).

As shown in Fig 7, phyla Spirochaetes, Firmicutes, Tenericutes, and Chloroflexi were significantly more abundant in diseased oysters than in healthy oysters in the hemolymph, while phylum Spirochaetes was more abundant in diseased oysters than in healthy oysters in the mantle (Wilcoxon signed-rank test, *p*<0.05). *Spirochaetes* was prevalent in both hemolymph and mantle in diseased pearl oysters.

**Fig 8:**
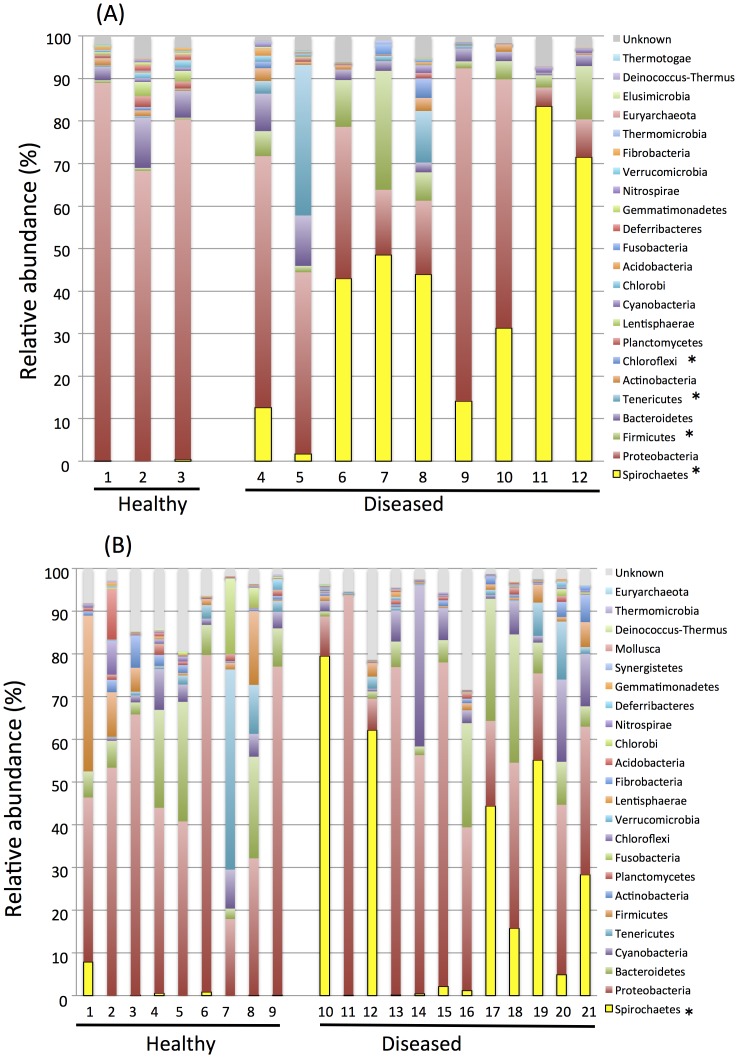
Relative abundance of microbial communities in (A) hemolymph and (B) mantle of healthy and diseased individuals to phylum level.

Stacked bar charts were generated to represent the proportion of sequences classified to phylum level taxa in hemolymph (Fig 8A) and mantle samples (Fig 8B) from healthy and diseased oysters (Table 1). In the hemolymph, *Proteobacteria* was dominant in healthy pearl oysters, while *Spirochaetes* was dominant in five of nine diseased pearl oyster samples. *Spirochaetes* was found in all diseased oyster samples, and it was also found in two of three healthy oyster samples. In the mantle, *Proteobacteria* was dominant in healthy pearl oysters, except for sample M7. In diseased pear oysters, *Spirochaetes* was present in all diseased oyster samples and was dominant in four of twelve samples. It was also present in six of nine healthy oyster samples.

After assembling 16S rRNA reads assigned to phylum Spirochaetes, 98.5% of the total analyzed reads were mapped to a single contig, named C10 (S3 Table), for which the sequence is available in the S1 File. The reads that form C10 were derived from all diseased pearl oyster samples. No reads derived from healthy pearl oysters were mapped to contig C10.

### 16S rRNA gene sequencing and phylogenetic analysis

**Fig 9:**
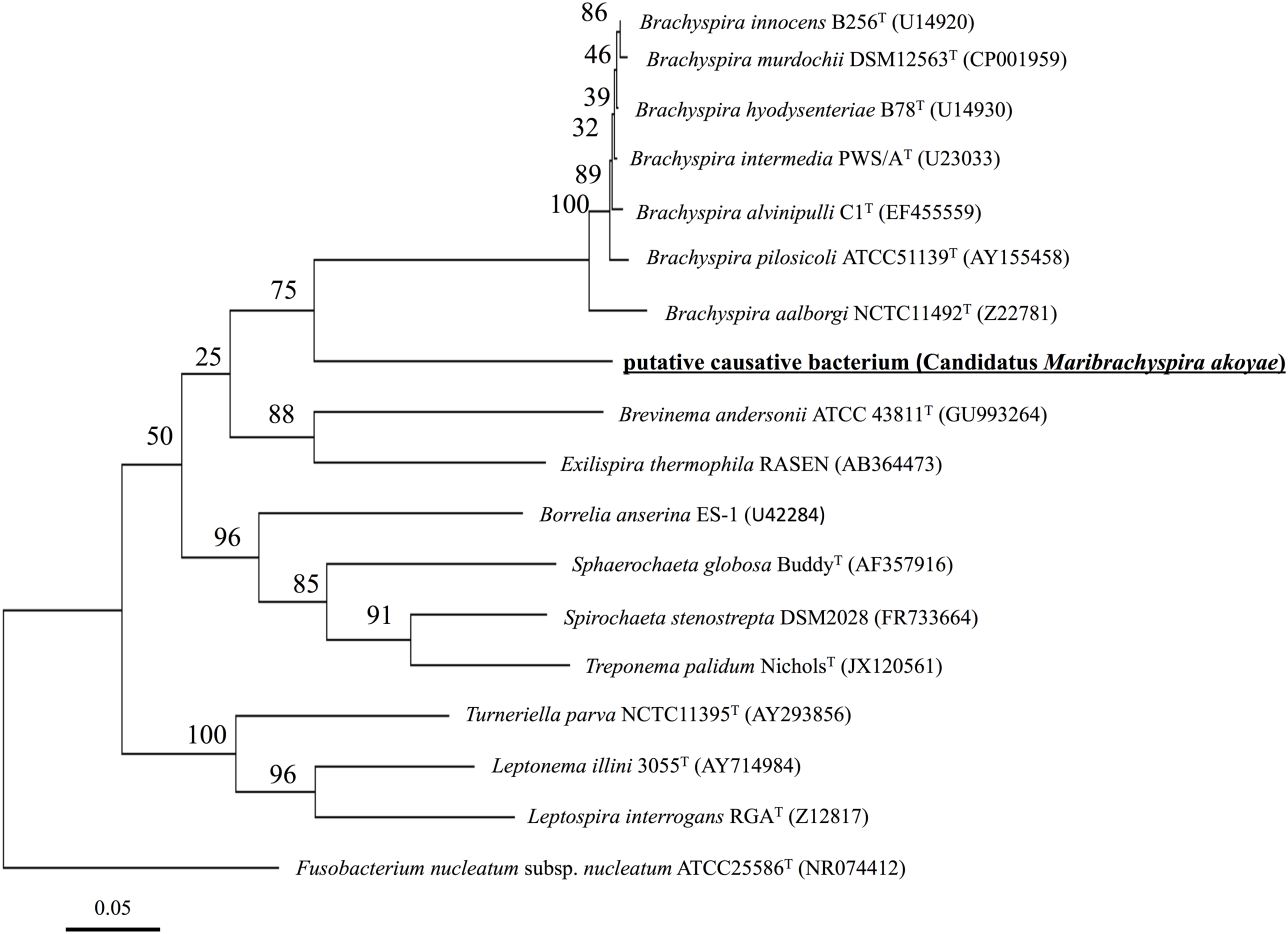
Phylogenetic tree for 16S rRNA gene sequences of putative causative agent and those of representative type species of phylum Spirochaetes. Strain names and accession numbers of the 16S rRNA gene sequences follow the species name. The tree was constructed using the maximum-likelihood algorithm. Numbers at nodes represent the confidence limits estimated based on 1000 bootstrap replicates. The 16S rRNA gene sequence of *Fusobacterium nucleatum subsp*. *nucleatum* ATCC25586^T^ was used to root the tree. Bar, 0.05 *K*nuc in nucleotide sequences.

Among 48 sequenced plasmid clones, 5’ sequences of 8 clones were found to be overlapping with the contig C10 sequence by alignment analysis using MEGA7. No single nucleotide difference was observed in full-length sequences among these eight clones. Phylogenetic analysis of near full-length 16S rRNA sequences of the putative causative bacterium and those of other Spirochaetes genera revealed that genus *Brachyspira* was most closely related, but the putative causative bacterium was distinct from the clade containing the type species of genus *Brachyspira* (Fig 9). The low similarity of the 16S rRNA sequence of the putative causative bacterium with its phylogenetically close neighbors in genus *Brachyspira* is sufficient to suggest that the bacterium represents a novel species in a new genus of a novel family within the order *Spirochaetales*, class *Spirochaetes*, phylum ‘*Spirochetes*’.

### FISH and fluorescent immunostaining

**Fig 10:**
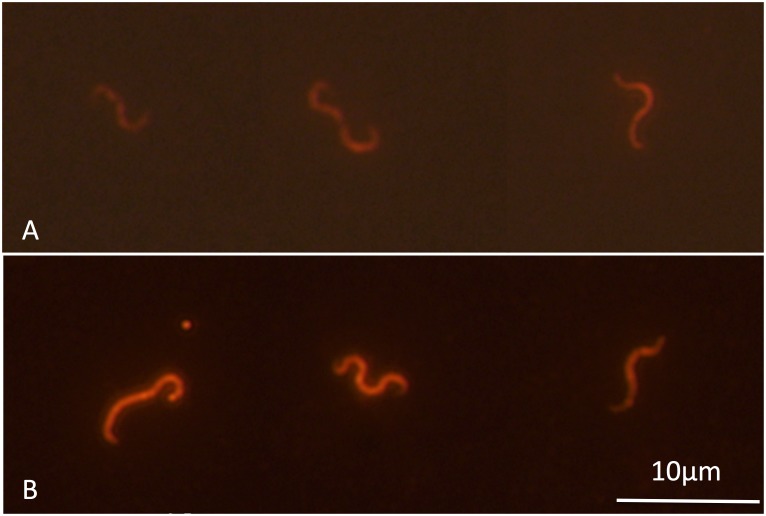
Fluorescence micrographs showing specific detection of bacteria cells from diseased pearl oyster hemolymph after (A) *in situ* hybridization and (B) fluorescence immunostaining.

The morphologically *Brachyspira*-like spirochetes were observed by FISH using the AOD-Cy3 probe and immunostaining using *B*. *aalborgi* and *B*. *pilosicoli* antiserum in three diseased pearl oysters (Fig 10). No spirochetes were detected in the smears from healthy pearl oysters. No staining of the bacterium was observed in FISH using non-AOD-Cy3 probe and immunostaining using *T*. *pallidum* antisera and naive rabbit serum.

## Discussion

Spirochetes had been detected in crystalline styles of bivalves [30–33]and gastropods [34]. 16S rRNA sequences, which are affiliated with *Brachyspira*, were also reported from the crystalline style of *Arctica islandica* [33]. Spirochetes found in the crystalline styles of bivalves are thought to be commensal organisms [34, 35], but their involvement with disease has never been discussed. The present study clearly indicates the possible involvement of Spirochaetes in AOD.

The bacterium most closely related to genus *Brachyspira* was identified as the putative causative agent of AOD based on the following evidence: 1) reads identified by BLAST as bacteria sequences were dominant in shotgun metagenome analysis, while few animal virus sequences were found; 2) penicillin administration was effective for blocking the occurrence of AOD; and 3) 16S rRNA sequences, which are closely related to genus *Brachyspira*, were detected from all diseased pearl oysters, while on the other hand, no such sequences were detected in healthy pearl oysters; and 4) morphologically *Brachyspira*-like cells were observed in diseased pearl oysters by FISH and immunocytochemistry using *Brachyspira* antiserum, but not in healthy pearl oysters.

Despite attempts to identify the causative agent of AOD since 1999, it had not yet been identified. The main reason for the lack of success in identifying the causative agent is the presence of a wide variety of microorganisms in the body of the pearl oyster. Hemolymph of healthy marine bivalves contains various bacteria [36–40] and the pearl oyster is no exception, and some viruses have also been reported in AOD-affected pearl oysters [3, 41]. In our previous shotgun cloning analysis with Sanger sequencing, sequences homologous to *Nanovirus*, *Geminivirus*, and *Circovirus* were detected in pearl oysters (unpublished data). Several kinds of bacteria were also identified in the sequences of 16S rRNA clones (unpublished data). The read depth for traditional cloning-dependent sequencing methods was too low to provide an overview of the complex microbiota in pearl oysters because new microorganisms were found with every new round of analysis. Therefore, we applied next-generation sequencing technology for metagenomic analysis to the survey of potential causative agents of AOD.

Few animal virus sequences were obtained by shotgun metagenomic analysis. The most abundant animal virus was *Flaviviridae*, a single-stranded RNA virus. Detecting a non-retroviral RNA virus sequence from a genomic sample is puzzling, but as reported in insects [42–44], *Flaviviridae* RNA sequences may have been integrated in the form of DNA in the pearl oyster genome. As for DNA viruses, large dsDNA viruses such as *Mimiviridae*, *Megaviridae*, *Herpesviridae*, *Baculoviridae*, *Poxviridae*, and *Polydnaviridae* are dominant among animal viruses. Large viruses might preferentially sediment during ultracentrifugation at 20,000×g for 1 h. In contrast to viruses, bacteria sequences accounted for three-quarters of the total read number. Therefore, we hypothesize that the causative agent of AOD is a eubacterium. This hypothesis was supported by the results of penicillin administration test because AOD did not occur in penicillin-administered groups.

The causative agent of AOD was reported to be abundant in the mantle based on transplantation experiments of pieces of various tissues from diseased pearl oysters into healthy pearl oysters [12]. Thus, the mantle was chosen as an additional target organ of analysis along with the hemolymph. As has been reported in oysters [39, 45], bacterial communities in healthy pearl oysters were dominated by the phylum Proteobacteria. On the other hand, the occurrence of the phylum Spirochaetaceae was prominent in diseased pearl oysters. But it was also detected in some healthy pearl oysters. Thus, 16S rRNA sequences assigned to the phylum Spirochaetes were assembled in order to analyze the variety of Spirochaetes in pearl oyster. Assembling the Spirochaetaceae reads elucidated three findings: 1) of the 38 contigs, a contig named C10 was found only in diseased pearl oyster, 2) contig C10 is present in all diseased pearl oyster samples, and 3) reads that form contig C10 are highly dominant among the reads assigned to phyla Spirochaetes. These three findings suggest that a bacterium of phylum Spirochaetaceae, the origin of the C10 sequence, is the most likely candidate as the causative agent of AOD. As later elucidated, the near full-length 16S rRNA sequence of C10 was most closely related to members of the family *Brachyspiraceae*. Therefore, a bacterium closely related to *Brachyspiraceae* family is the most reasonable candidate for the etiological agent. This conclusion is supported by the observation of morphologically *Brachyspira*-like bacteria in the smear of the diseased pearl oysters hemolymph, but not in that from healthy pearl oyster hemolymph.

Do the known features of the causative agent of AOD match the characteristics of Spirochaeta? The features of the causative agent of AOD are 1) passes through 0.45 μm filter (S1 Table), 2) pathogenicity is inactivated by immersion in PBS (unpublished), and 3) penicillin susceptible (this study). Spirochaeta is known to pass through a 0.1 μm [46] or 0.2 μm filter [47]. The body of the Spirochaeta is surrounded by an envelope and is fragile when subjected to mechanical stress [48–50]. Members of *Treponema*, a genus of phylum Spirochaetes, change their spirochetal morphology to spherical in low osmotic pressure as the result of degeneration [51]. Penicillin has been used to treat spirochaetosis due to causative organisms such as *Treponema* [52], *Leptospira* [53], *Borrelia* [54], and *Brachyspira* [55]. Thus, even if Spirochaetes is assumed to be the causative agent of AOD, there are no discrepancies because the features of the causative agent of AOD and the features of Spirochaetes are similar. However, further study, such as infection experiment using isolated bacteria, is needed to define a causal relationship between Spirochaetes and AOD. However, all attempts to isolate the bacterium in pure culture were unsuccessful.

This is the first report of applying metagenomic analysis to the search for the causative agent of a shellfish disease. Due to the presence of various bacteria in the body of the shellfish, identification of pathogenic bacteria by traditional culture-dependent methods is difficult. Further, the lack of marine mollusk cell lines and the lack of an antibody-producing system in invertebrates makes the identification of a pathogenic organism very difficult [56]. Metagenomic analysis makes it possible to assay the microbial flora broadly using a culture-independent method. Thus, metagenomics is an effective tool for the study of shellfish diseases.

Using short sequence obtained by metagenomic analysis as a clue, the near full-length 16S rRNA sequences of the putative causative agent was cloned and phylogenetic analysis was conducted. The bacterium was related most closely to members of the family *Brachyspiraceae*, but it was distinct from the clade containing the type species of the family *Brachyspiraceae*. Therefore, we propose ‘Candidatus Maribrachyspira akoyae’ gen. nov, sp nov., as the identification of the putative causative agent of AOD in pearl oyster.

### Description of ‘ Candidatus *Maribrachyspira akoyae* gen. nov., sp nov.’

In accordance with guidelines for description uncultivated organisms [57], we propose giving this causative agent of AOD a *Candidatus* designation ‘Candidatus *Maribrachyspira akoyae*’ (Mari.bra.chy.spi.ra; L. n. *mare* sea; Gr. adj. *brachys*, short; L. fem. n. *spira*, a coil, spiral; N.L. fem. n. *Maribrachyspira*, marine short spiral; akoyae, Japanese name of a pearl oyster, pertaining to the name of the host animal). The short description is as follows: genus *Brachyspira*-like morphology; 5.4–11.8×0.41–0.78 μm in size; basis of assignment, 16S rDNA sequence accession number LC212988, 16S rRNA-targeted nucleotide probe (5′-GTATTAATCCAATTTTCACT-3′); antisera to *B*. *aalborgi* and *B*. *pilosicoli* cross-reactive but not antisera to *T*. *pallidum*; putative pathogen of pearl oyster, *Pinctada fucata martensii*.

## Supporting information

S1 Table Pathogenicity of filtrates. (XLSX)

S2 Table Primers used for 16S rRNA gene sequencing (5′–3′). (XLSX)

S3 Table Assembly of 16S rRNA reads assigned to phylum Spirochaetes. (XLSX)

S1 File Contig C10 sequence. (DOCX)
