# Supplementary material for: A Spirochaete is suggested as the causative agent of Akoya oyster disease by metagenomic analysis
Source: PLoS One. 2017 Aug 3;12(8):e0182280. doi: 10.1371/journal.pone.0182280 (PMC5542438; doi:10.1371/journal.pone.0182280)
Supplement: S1 File — (DOCX) [file pone.0182280.s004.docx]

Supplemental data 1

Contig C10 sequence.

CGAGAGATACGAGTTTGATCATGGCTCAGAATGAACGCTGGTGGTATGTTTTAAGCATGCAAGTCGAACGAAGATAATTACGCTTGTATGAGTGTGATTAGACTGAGTGGCGAACGGGTGAGTAACGCGTAAATAATCTGCCTTAAAGACTGGGATAGCCTAGTGAAAATTGGATTAATACCGGATGTATTCATAAAAATATAGGTTTTTATGAAAAAAGGAGCAATCCGCTTTAAGATGAGTTTGCGTTCTATTAGCTTGTTGGTGGAGTAAAAGCCTACCAAGGCTACGATAGATAGCCGGCCTGAGAGGGTGTACGGCCACATTGGGACTGAGATACGGCCCAGACTTCTACGGAAGGCAGCAGCTAAGAATATTCCGCAATGGGCGAAAGCCTGACGGAGCAATACCACGTGAATGATTAAGGCCTGAAAGGGTTGTAAAGTTCTTTTATTGAGGAAGAATAAAGTGACGGTACTCAATGAATAAGTTCCGGCTAATTACGTGCCAGCAGCCGCGGTAAAGTATCTCTCGCTAAGACTGGGATAGCCTAGTGAAAATTGGATTAATACCGGATGTATACAAT
